# AI generated annotations for Breast, Brain, Liver, Lungs, and Prostate cancer collections in the National Cancer Institute Imaging Data Commons

**DOI:** 10.1038/s41597-025-05666-6

**Published:** 2025-07-29

**Authors:** Gowtham Krishnan Murugesan, Diana McCrumb, Rahul Soni, Jithendra Kumar, Leonard Nuernberg, Linmin Pei, Ulrike Wagner, Sutton Granger, Andrey Y. Fedorov, Stephen Moore, Jeff Van Oss

**Affiliations:** 1 BAMF Health, Grand Rapids, MI USA; 2 Brigham and Women’s Hospital and Harvard Medical School, Boston, MA USA; 3 Frederick National Laboratory for Cancer Research, Frederick, MD USA; 4 National Institute of Health, Bethesda, MD USA

**Keywords:** Image processing, Translational research

## Abstract

The Artificial Intelligence in Medical Imaging (AIMI) initiative aims to enhance the National Cancer Institute’s (NCI) Image Data Commons (IDC) by releasing fully reproducible nnU-Net models, along with AI-assisted segmentation for cancer radiology images. In this extension of our earlier work, we created high-quality, AI-annotated imaging datasets for 11 IDC collections, spanning computed tomography (CT) and magnetic resonance imaging (MRI) of the lungs, breast, brain, kidneys, prostate, and liver. Each nnU-Net model was trained on open-source datasets, and a portion of the AI-generated annotations was reviewed and corrected by board-certified radiologists. Both the AI and radiologist annotations were encoded in compliance with the Digital Imaging and Communications in Medicine (DICOM) standard, ensuring seamless integration into the IDC collections. By making these models, images, and annotations publicly accessible, we aim to facilitate further research and development in cancer imaging.

## Background & Summary

The expanding availability of curated medical imaging datasets and the rapid progress in deep learning (DL) have yielded state-of-the-art algorithms for tumor and organ segmentation, yet their translation to routine clinical practice remains limited by three data-centric bottlenecks. First, a systematic review^1^ of 110 publicly released MRI collections—encompassing ~1.7 million examinations—showed that only a small fraction provides voxel-level annotations or harmonized metadata, restricting their usefulness for supervised model development. Second, the AI for Health Imaging (AI4HI) network^2,3^ has underscored the need for a unified, DICOM-compliant metadata schema to guarantee interoperability across institutions and downstream analytical pipelines. Third, the same consortium stresses that reliable decision-support tools require rigorous data-lineage documentation, multi-institutional external validation, and post-deployment performance monitoring. In addition, one of the most critical barriers to assembling large, well-annotated, widely representative medical-image datasets is the set of constraints imposed by data security, patient privacy, interoperability, and appropriate data-use agreements^1,4^. In the present work, these challenges are addressed through the use of de-identified data from the secure, cloud-hosted Imaging Data Commons infrastructure, the adoption of DICOM-native metadata standards, and the distribution of containerized training and inference environments that run reproducibly on both local and cloud infrastructure.

To close these gaps within the Imaging Data Commons (IDC)—where many collections still lack reliable tumor, organ, or tissue labels—we (i) trained a suite of nnU-Net models on publicly available CT and MRI cohorts covering cancers of the breast, chest, kidney, prostate, and liver; (ii) generated DICOM-SEG masks for 11 IDC collections, expanding our previous release^5^ from 1,925 to more than 3,500 studies; and (iii) released all pre-trained weights, and Docker containers under permissive licenses. A stratified subset of the automated segmentations was reviewed and corrected by a board-certified radiologist to quantify annotation quality. All images, masks, and accompanying clinical descriptors adhere to a FAIR-compliant, DICOM-based metadata structure, enabling seamless ingestion into other repositories and facilitating future multi-institutional validation studies. All models, images, and annotations are publicly accessible, supporting further research and development in cancer imaging.

## Methods

In this project, we developed nnU-Net models to generate AI segmentations for six distinct tasks: brain, breast, lung, liver, prostate, and kidney organ and tumor segmentation. These tasks utilize 11 unique IDC collections, as outlined in Table 1. New models were trained for four tasks: brain, breast, liver, and lung, using publicly available datasets detailed in Table 1. For the kidney and prostate segmentation tasks, we employed models developed as part of previous work to enrich additional IDC collections with AI assisted annotations.

**Table 1:** IDC collections Enriched with AI-assisted annotations: Summarizes IDC collections, detailing the number of studies, imaging modalities of interest (MOI), curated studies enriched with AI annotations, specific annotations, training data for each task, and the number of studies validated by radiologists.

Task	IDC Collections Enriched (Studies)	MOI	Curated Images with MOI	Segments	Training Data/ Model (Dataset Size)	Images validated by Radiologists
**Brain-MR**	UPENN-GBM (630)^24^	MR (T1, T2, FLAIR, T1c)	541	Whole Tumor, Enhancing Tumor, Non-Enhancing Tumor	Brats2021^7,8^ (1251)	45
**Breast-MR**	Duke-Breast-Cancer-MRI (922)^11^	MR (T1 post contrast)	805	Breast, FGT, and Tumor	TCIA-ISPY1-Tumor-SEG-Radiomics^10^ (98), Duke-Breast-Cancer-MRI-Supplement-v3^11^ (489)	92
**Kidneys-CT**	TCGA-KICH (12)^25^, TCGA-KIRP (23)^26^, and CPTAC-CCRCC (57^27^)	CT (Post contrast)	64	Kidneys, Cysts, Tumors	Kidney-CT^18^ BAMF-AIMI model	7
**Lung CT**	QIN Lung CT (47)^28^, SPIE AAPM Lung CT Challenge (70)^29^, and National Lung Screening Trial (1000)^30^	CT	1137	Lungs nodules	NSCLC Radiomics^17^ (416), LIDC_IDRI^31^ (883),	114
**Liver-CT**	HCC_TACE_Seg (105)^32^, Colorectal-Liver-Metastases(197)^33^	CT	515	Liver, Tumors	Medical Decathlon Dataset^14^ (131), LiTS 2017^13^ (131)	52
**Prostate-MR**	Prostate MRI US-Biopsy (842)^34^	MR (T2)	817	Prostate	Prostate-MR^19^ BAMF AIMI model	81

### Data curation

All source datasets were fully de-identified under their respective open licenses. No additional Institutional Review Board approval was required for the present study. For each task, source radiological images from publicly available NCI IDC collections were selected using BigQuery commands and then downloaded; the code is made available in Zenodo (Table 6). These images were filtered to match the modality of interest requirements (Table 1) for each specific task based on the model inputs. Given the large size of the National Lung Screening Trial, 26408 cases, a subset of 1042 was selected using the query specified by Krishnaswamy *et al*.^6^, to include only CT scans of subjects who were clinically confirmed positive for lung cancer. Detailed information on the modalities of interest for each task, along with the number of curated images, is listed in Table 1.

### Model training and data processing

#### Brain tumor segmentation

We used the BRATS 2021^7,8^ dataset, which includes 1251 paired T1, T1 post-contrast, T2, and FLAIR images, to segment edema, tumor core, and enhancing tumor regions to train an nnUNet model (Brain MR). To improve model generalizability, we permuted the order of the input MRI sequences during training. DICOM images downloaded from IDC were converted into the Neuroimaging Informatics Technology Initiative (NIfTI) format, then all the selected MR contrasts images were reoriented to RAS, N4 bias corrected, registered to T1c, skull stripped, and then registered to MNI^9^ template before deriving AI annotations of tumor components using Brain-MR model. The AI derived annotations were post processed,reoriented and inverse registrated with T1c to get them back to each of MR contrast image spaces. The AI derived annotation in MNI space with different permutations of input order was compared with automatic annotations in the UPENN-GBM dataset (Supplementary Table 1).

#### Breast, fibroglandular tissue, and tumor segmentation

The nnUNet model was trained using 489 post-contrast T2 images from the Duke-Breast-Cancer-MRI-Supplement-v3^10^ dataset. For breast tumor segmentation, an additional nnUNet model was trained using 98 post-contrast T2 MR images from the TCIA-ISPY1-Tumor-SEG-Radiomics^11,12^ dataset. Tumor segmentations from this dataset include both enhancing and non-enhancing tumor regions, defining the structural tumor volume (STV). DICOM images downloaded from IDC were converted into NIfTI images and used as input to the Breast-MR model to generate AI Annotations. Predictions outside the breast were removed using connected component analysis. The output from each model was combined to create a single segmentation output with three labels: breast, fibro glandular tissue (FGT), and tumor.

#### Liver and tumor segmentation

CT images from the LiTS 2017^13^ and Medical Decathlon datasets^14^ were used to train the nnUNet model for liver and tumor segmentation. We utilized selected TotalSegmentator^15^ outputs to develop an anatomically informed model. The liver-CT model was trained to predict liver and liver tumors, as well as other abdominal organs, including the duodenum, gallbladder, intestines, kidneys, lungs, pancreas, and spleen.

#### Lung and nodules segmentation

The model for lung and nodules segmentation was trained using 883 CT images from the LIDC-IDRI^16^ dataset and 416 CT images from the NSCLC Radiomics^17^ dataset, each annotated for lung lesions and nodules. Annotations for the lung regions in the training dataset were generated by TotalSegmentator^15^. Predictions outside the Lungs were removed using connected component analysis. Nodules smaller than 3 mm or larger than 30 mm are removed from the predictions.

#### Kidney and prostate segmentation

For kidney tumors and cysts and prostate segmentation, we utilized the kidney-CT^18^ and prostate-MR^19^ models from the BAMF AIMI project.

Details on the training data, input image types, and output segmentation for each model are provided in Table 1. Comprehensive information on the training, pre-processing, and post-processing procedures is available on Zenodo (Table 6)

### Quality assessment

To ensure the quality of AI-generated annotations, approximately 10% of these annotations were validated by certified radiologists due to limited budget(Table 1). Quality metrics, including the Dice coefficient, normalized surface distance (NSD), and detection accuracy, were reported. We have provided code for reproducibility, calculating quality metrics, and enabling the downloading of data from IDC (Table 6).

To control project costs, the radiologists agreed to review and correct AI generated segmentations based on an estimation of the time required, not the actual time spent. Every organ-specific dataset was independently assessed by subspecialty radiologists with extensive clinical experience. Two fellowship-trained neuroradiologists with 14 and 18 years of practice, respectively, evaluated the brain MRI masks. An abdominal radiologist with 13 years of experience reviewed the liver segmentations. Breast labels were validated by two dedicated breast radiologists with 18 and 30 years of experience, while three radiologists with 16, 20, and 15 years of experience, respectively, assessed the lung masks. Finally, an additional radiologist with 20 years of expertise cross-checked the kidney annotations. These measures enhance the robustness of the quality-control workflow and are detailed in the revised manuscript.

**Fig. 1:**
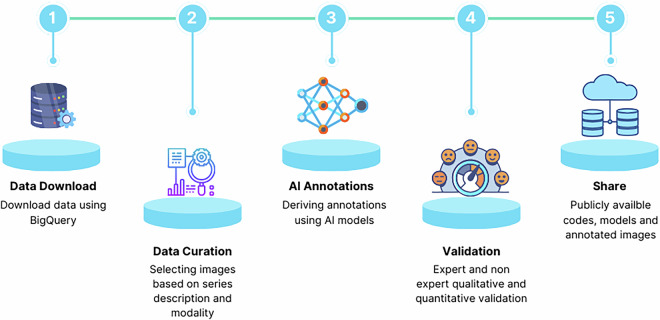
The AI in Medical Imaging (AIMI) workflow: Describes overall workflow from Data Download, Data Curation, Annotations, Validation and Sharing of AI annotation dataset and model weights.

**Fig. 2:**
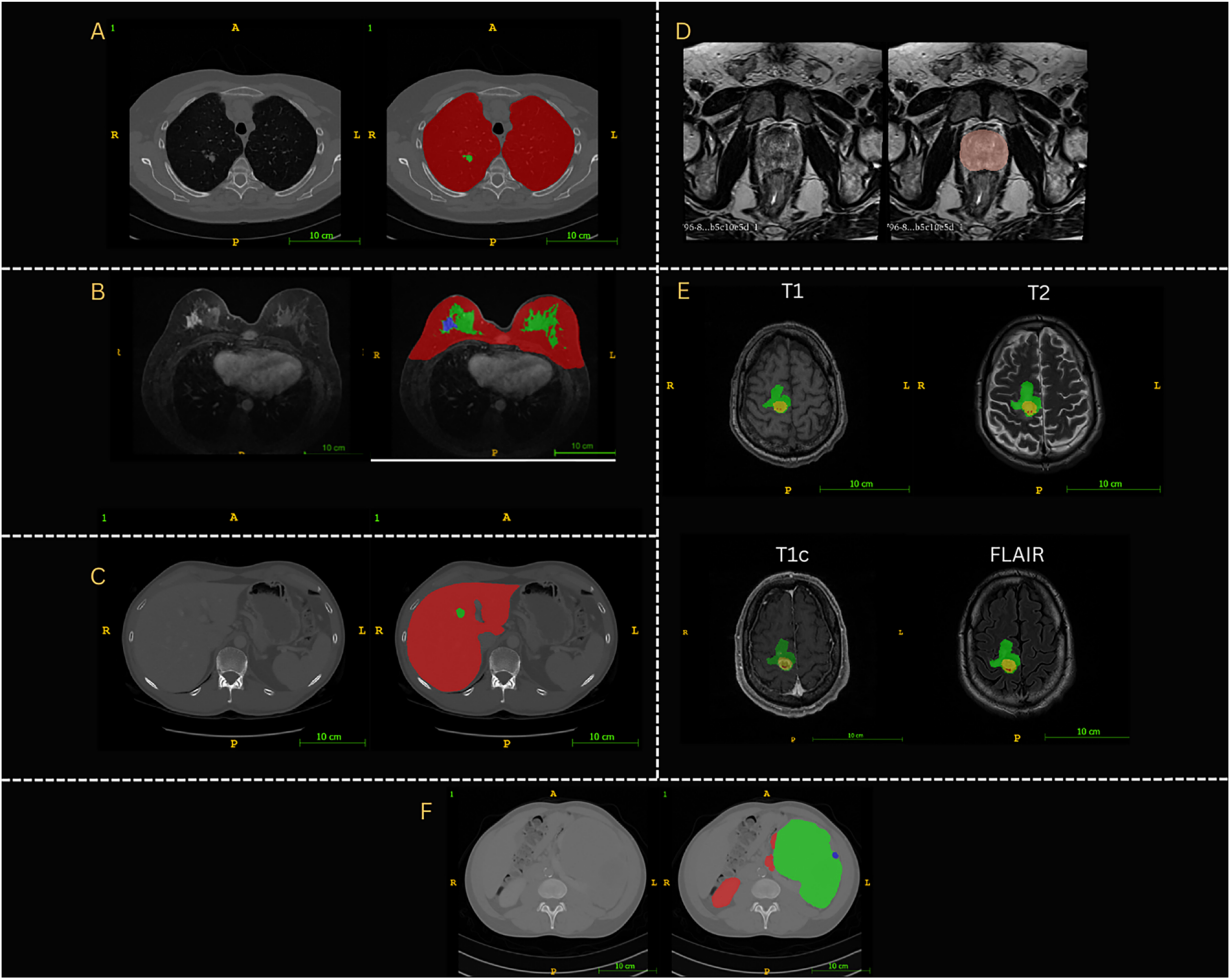
AI Annotations: Representations of input images and AI annotations for (**A**) Lungs and nodules, (**B**) Breast, FGT and Tumor, (**C**) Liver and Tumor, (**D**) Prostate, (**E**) Brain Tumor components (Edema, Enhancing and Non enhancing) and (**F**) Kidneys, Cysts, and Tumor.

The Radiologists used 3DSlicer to load the images and segmentations. For all tasks except Brain, the scans were loaded from DICOM format. For the Brain task, the scans were loaded from the *NIfTI* formatted files after they had been pre-processed for skull stripping and co-registration. The AI segmentation files were loaded from NRRD formatted files. The radiologists reviewed and edited the segmentations as needed to ensure accuracy and quality. After making corrections, they returned the updated segmentations in NRRD format. These corrected segmentations were subsequently converted to DICOM-SEG format for inclusion in the IDC. For the Brain task, segmentations were transformed back to the coordinate space of each of the original scans before conversion to DICOM-SEG. The overall workflow of our analysis is illustrated in Fig. 1, and the representation of AI annotations generated is shown in Fig. 2.

## Data Records

The reviewers scoring and comments, as well as DICOM Segmentation objects for the AI predictions and reviewer’s corrections, are available in Zenodo^20^ (https://zenodo.org/records/13244892).

Each zip file in the collection correlates to a specific segmentation task. The standard folder structure is as follows:

- *ai-segmentations-dcm* This directory contains the AI generated segmentations in DICOM-SEG format for all analyzed IDC collection files.
- *qa-segmentations-dcm* This directory contains manually corrected segmentation files based on the AI prediction in DICOM-SEG format. Only a fraction, ~10%, of the AI predictions were corrected. A radiologist performed corrections to the AI model segmentations.
- *qa-results.csv* CSV file linking the study/series UIDs with the AI segmentation file, radiologist corrected segmentation file and radiologist ratings of AI performance. Reviewer Likert scores and review comments for the segmentations are also included in this file. (Tables 2 and 3)

**Table 2:** Common columns and their descriptions for qa-results.csv files.

Column	Description
**Collection**	The name of the IDC collection for this case
**PatientID**	PatientID in DICOM metadata of scan. Also called Case ID in the IDC
**StudyInstanceUID**	StudyInstanceUID in the DICOM metadata of the scan
**SeriesInstanceUID**	SeriesInstanceUID in the DICOM metadata of the scan
**Validation**	true/false if this scan was reviewed by a radiologist
**Reviewer**	Coded ID of the reviewer. Radiologist IDs start with ‘rad’ non-expect IDs start with ‘ne’
**AimiProjectYear**	2023 or 2024, This work was split over two years. The main methodology difference between the two is that in 2023, a non-expert also reviewed the AI output, but a non-expert was not utilized in 2024.
**AISegmentation**	The filename of the AI prediction file in DICOM-seg format. This file is in the ai-segmentations-dcm folder.
**CorrectedSegmentation**	The filename of the reviewer corrected prediction file in DICOM-seg format. This file is in the qa-segmentations-dcm folder. If the reviewer strongly agreed with the AI for all segments, they did not provide any correction file.
Was the AI predicted * label accurate?	This column appears one for each segment in the task. The reviewer rates that segment quality on a Likert scale
**Do you have any comments about the AI predicted ROIs?**	Open ended question for the reviewer
**Do you have any comments about the findings from the study scans?**	Open ended question for the reviewer

Some columns in qa-results.csv are answers to questions posed to the reviewers. These questions were specific to the task the CSV file refers to. For example, “Was the AI predicted Cyst label accurate?” was asked for the kidney task, but variations asking about each segment were asked about every task

**Table 3:** Likert Score description used by reviewers to assess the quality of the AI annotations per case in the Validation set.

*Likert Score*	Description
**Strongly agree**	Use-as-is (i.e., clinically acceptable, and could be used for treatment without change)
**Agree**	Minor edits that are not necessary. Stylistic differences, but not clinically important. The current segmentation is acceptable.
**Neither agree nor disagree**	Minor edits that are necessary. Minor edits are those that the review judges can be made in less time than starting from scratch or are expected to have minimal effect on treatment outcome.
**Disagree**	Major edits. This category indicates that the necessary edit is required to ensure correctness, and sufficiently significant that user would prefer to start from the scratch.
**Strongly disagree**	Unusable. This category indicates that the quality of the automatic annotations is so bad that they are unusable.

The DICOM segmentation files contain self-describing information in the metadata.

- Each segmentation can be linked back to the original DICOM scans from the DICOM metadata in each segmentation file. The DICOM data element SeriesInstanceUID (0020,000E) in the data element ReferencedSeriesSequence (0008,1115) refers to the original DICOM scan this segmentation was derived from.
- The SegmentSequence (0062,0002) contains metadata about each of the segments such as:
  - SegmentNumber (0062,0004) - The numerical value of pixels that comprise this segment
  - SegmentDescription (0062,0006) – A human readable description of the segment
  - SegmentAlgorithmType (0062,0008) – Either *AUTOMATIC* for AI model outputs or SEMIAUTOMATIC for manual reviewer corrections of the AI output.

The DICOM segmentations have been integrated into the IDC. From that portal, it is possible to view the segmentations overlayed on the images from which they were derived. The direct link to the segmentation collection is in IDC^21^ (https://portal.imaging.datacommons.cancer.gov/explore/filters/?analysis_results_id=BAMF-AIMI-Annotations).

## Technical Validation

The AI models were evaluated on the following series of metrics. Some of these were only applicable to a subset of the model tasks.

- Sørensen–Dice coefficient^22^ (DSC): measures the similarity between volumetric segmentations, ${V}_{A}$ and ${V}_{B}$. It is twice the intersection of the volumes over the sum of the volumes.
  $${DSC}=\frac{2({V}_{A}\cap {V}_{B})}{{V}_{A}+{V}_{B}}$$
- Normalized Surface Dice^23^ (NSD): measures surface distance similarity. It measures the amount of the surface of a volume(${S}_{A}$) that is within a tolerance ($\tau$) of the surface of another volume (${S}_{B}^{\tau }$). This is calculated for both surfaces and normalized to the total surface of the volumes. NSD tolerance level for each task were selected based on the acceptable error for the segmentation provided by Antonelli M *et al*.^14^.
  $${NSD}=\frac{\left({S}_{A}\cap {S}_{B}^{\tau }\right)+({S}_{B}\cap {S}_{A}^{\tau })}{{S}_{A}+{S}_{B}}$$
- 95% Hausdorff Distance: measures surface agreement. It is the distance at which 95% of the points on Surface A have a point on Surface B less than it.

For each task, 10% of the images enriched for IDC imaging collections were evaluated and corrected by expert radiologists. The number of images evaluated, along with the Dice coefficient, 95% Hausdorff distance, and normalized surface distance for each of the model outputs for each task, are detailed in Table 4. The higher Dice scores observed in certain tasks, such as Kidneys-CT, may be attributed to experts not fully correcting the AI-generated annotations. This may reflect correction bias, as experts were refining AI-derived segmentations rather than generating them independently from scratch. In addition to the quantitative evaluation results, Table 5 lists the Likert score ratings for each task. Across the validated scans within each sub task, radiologist majority of them Use as is or minor edits that are not necessary which reflects on the efficienty of the models perfromance.

**Table 4:** Quantitative Analysis: Quantitative metrics annotations for each task.

Model	Segmentations	Dice	95% Hausdorff Distance	NSD ^(tolerance(mm))^
**Brain-MR**	Whole Tumor	0.98 ± 0.07	6.88 ± 0.34	0.98 ± 0.04^2^
	Enhancing Tumor	0.95 ± 0.13	6.57 ± 0.24	0.99 ± 0.03^2^
	Non enhancing Tumor	0.97 ± 0.08	0.42 ± 1.05	0.97 ± 0.09^2^
**Breast-MR**	Breast	0.99 ± 0.01	0.74 ± 2.92	0.09 ± 0.22^3^
	Fibroglandular Tissue	0.80 ± 0.29	8.75 ± 12.92	1.82 ± 4.17^2^
	Lesions	0.57 ± 0.36	41.44 ± 51.78	9.36 ± 13.79^2^
**Kidneys-CT**	Kidneys	1.0 ± 0.0	0.00 ± 0.00	0.00 ± 0.00^3^
	Cysts	1.0 ± 0.0	0.00 ± 0.00	0.15 ± 0.38^2^
	Tumors	1.0 ± 0.0	0.00 ± 0.00	0.00 ± 0.00^2^
**Lung-CT**	Lungs	1.0 ± 0.0	0.00 ± 0.00	0.02 ± 0.11^2^
	Nodules	0.78 ± 0.28	62.07 ± 10.54	10.54 ± 14.43^2^
**Liver-CT**	Liver	0.99 ± 0.02	2.33 ± 7.70	0.29 ± 0.95^7^
	Tumors	0.80 ± 0.35	19.73 ± 38.35	4.38 ± 8.70^2^
**Prostate-MR**	Prostate	0.99 ± 0.02	1.07 ± 1.24	0.15 ± 0.18^4^

NSD tolerance levels for each task were selected based on the acceptable error for the segmentation provided by Antonelli *et al*.^14^.

**Table 5:** Qualitative Analysis: Radiologist’s Likert score ratings for validation dataset.

Task	Label	Strongly disagree	Disagree	Neither agree nor disagree	Agree	Strongly agree
**Brain-MR**	**Necrosis**	0	0	8	4	36
	**Edema**	0	2	6	7	33
	**Enhancing Tumor**	0	4	13	2	29
**Kidney-CT**	**Kidney**	0	0	0	0	7
	**Tumor (Kidney)**	0	0	0	0	7
	**Cyst**	0	0	1	0	6
**Liver-CT**	**Liver**	1	3	2	5	36
	**Tumor (Liver)**	6	7	5	3	26
**Lung-CT**	**Lungs**	0	1	19	12	80
	**Lung Nodules (3 mm – 30 mm)**	0	13	29	28	42
**Prostate-MR**	**Prostate**	0	1	15	29	36

## Usage Notes

The AI models and datasets created in this project are intended for broad accessibility and straightforward replication within the research community. The trained nnU-Net model weights are hosted on Zenodo (https://zenodo.org/records/13244892), and Table 6 lists the precise download URLs. To foster reproducibility, we have posted comprehensive Jupyter notebooks in a public Git repository (Table 6); these notebooks detail every step of data processing, model training, and evaluation, allowing other investigators to validate and extend our workflow. The curated datasets, including the AI-generated labels and the radiologist-corrected annotations, are available through the National Cancer Institute’s Imaging Data Commons (IDC), ensuring that all underlying data can be readily accessed and reused.

**Table 6:** URLs for Model weights and git repositories.

Task	Model Weights	GitHub Repository
**Brain-MR**	10.5281/zenodo.11582627^35^	https://github.com/bamf-health/aimi-brain-mr
**Breast-MR**	10.5281/zenodo.11998679^36^	https://github.com/bamf-health/aimi-breast-mr
**Kidneys-CT**	10.5281/zenodo.8277846^18^	https://github.com/bamf-health/aimi-kidney-ct
**Lung CT**	10.5281/zenodo.11582738^37^	https://github.com/bamf-health/aimi-lung2-ct
**Liver-CT**	10.5281/zenodo.11582728^38^	https://github.com/bamf-health/aimi-liver-ct
**Prostate-MR**	10.5281/zenodo.8290093^19^	https://github.com/bamf-health/aimi-prostate-mr

Extensive documentation within the repository describes installation requirements and usage instructions, and it also provides examples that show how to apply the models to new datasets. To demonstrate the quality and reliability of the AI-generated annotations, we report evaluation metrics, including the Dice coefficient, normalized surface distance (NSD), and detection accuracy, which provide quantitative benchmarks for future comparisons. We actively welcome contributions from the research community: users may open issues, suggest improvements, and submit code through the Git platform. By sharing these resources and supporting materials, we aim to enhance the usability and reproducibility of our work, thereby accelerating progress in medical image analysis and AI research.

### Limitations

Several constraints of the present work should be acknowledged. Only 10% of the AI-generated segmentations were reviewed by a single board-certified radiologist; therefore, most masks remain unaudited, and inter-observer variability could not be accurately assessed. The models were not retrained after this quality check, so identified discrepancies did not inform further optimization. Performance was evaluated only on the CT and MRI datasets presented here, leaving generalisability to other imaging protocols or clinical settings untested. Future iterations should increase expert review coverage, incorporate multi-rater consensus, and integrate validation feedback into iterative model updates.

## Supplementary information

Quantitative Analysis: Evaluation of Brain-MR model on different possible permutation of input MR contrasts (T1,T2,FLAIR,and T1c)

## Data Availability

The AI model weights are accessible on Zenodo, and Jupyter notebook code to reproduce the analysis is available on Zenodo. The models have also been released on the https://MHub.ai platform. The URLs are provided in Table 6.
