# Supplementary material for: AI generated annotations for Breast, Brain, Liver, Lungs, and Prostate cancer collections in the National Cancer Institute Imaging Data Commons
Source: Sci Data. 2025 Jul 29;12:1317. doi: 10.1038/s41597-025-05666-6 (PMC12307902; doi:10.1038/s41597-025-05666-6)
Supplement: Supplementary file 1 — Quantitative Analysis: Evaluation of Brain-MR model on different possible permutation of input MR contrasts (T1,T2,FLAIR,and T1c) [file 41597_2025_5666_MOESM1_ESM.docx]

*Supplementary Table 1: Quantitative Analysis: Evaluation of Brain-MR model on different possible permutation of input MR contrasts (T1,T2,FLAIR,and T1c)*

|  | **WT** | **Edema** | **ET** | **NET** |
| --- | --- | --- | --- | --- |
| **Dice** | 0.97+/-0 | 0.93+/-0.1 | 0.81+/-0.2 | 0.92+/-0.1 |
| **Haussdorff** | 8.82+/-11.80 | 11.68+/-10.36 | 12.34+/-11.67 | 6.03+/-9.29 |
| **Jaccard Distance** | 0.06+/-0.05 | 0.13+-0.11 | 0.28+/-0.25 | 0.13+/-0.11 |
| **FPV** | 2.11+/-2.35 | 2.20+/-3.04 | 1.49+/-3.06 | 0.94+/-0.94 |
| **FNV** | 2.40+/-3.14 | 3.35+/-3.90 | 0.54+/-1.61 | 1.04+/-1.64 |
